# Native Arbuscular Mycorrhizal Fungal Communities in Trap Cultures Are Shaped by Traditional Host Plants and Agricultural Soils

**DOI:** 10.3390/jof11110792

**Published:** 2025-11-06

**Authors:** Michael Sakha, Joseph P. Gweyi-Onyango, Manoj Kaushal, Frederick P. Baijukya, Cargele Masso

**Affiliations:** 1 Department of Agricultural Science and Technology, Kenyatta University, Nairobi P.O. Box 43844-00100, Kenya; 2 Consultative Group on International Agricultural Research (CGIAR), International Livestock Research Institute (ILRI), Nairobi P.O. Box 30709-00100, Kenya; 3 Alliance of Bioversity International and International Center for Tropical Agriculture (CIAT), Duduville Campus Off Kasarani Road, Nairobi P.O. Box 823-00621, Kenya; m.kaushal@cgiar.org; 4 International Institute of Tropical Agriculture (IITA), Dar es Salaam P.O. Box 34441, Tanzania; f.baijukya@cgiar.org

**Keywords:** AM fungi propagation, landraces, monoculture, co-cultivation, semi-arid soils

## Abstract

Arbuscular mycorrhizal (AM) fungi inoculants are increasingly recognized as effective biofertilizers in sustainable agriculture. Typically, as a first step for AM fungi inoculum production, a trap culture system using mycotrophic host plants is commonly used to isolate AM fungi. However, the influence of traditional host plants and agricultural field soil on the composition of AM fungal communities in trap cultures remains poorly understood. The greenhouse study was conducted to assess the sporulation capacity of AM fungi by applying a trap culture technique using traditional varieties endemic to the semi-arid areas of eastern Kenya as host plants, along with soils sourced from the same area. The traditional varieties used included Kinyaanya maize, Vaasya sorghum, and Katumbuka beans. Soil samples were collected from 34 agricultural fields. Trap culture pots were established and maintained in a greenhouse for 120 days. The experiment was set up in a completely randomized design. AM fungi spores were extracted using the wet-sieving and decanting method, and healthy spores were selected for morphological analysis. Across all three host plants and the control (original agricultural field soils), six AM fungal genera were identified: *Acaulospora*, *Scutellospora*, *Gigaspora*, *Dentiscutata*, *Racocetra*, and *Funneliformis*. Our results demonstrated that traditional host plants differentially influence AM fungal sporulation. Notably, trap cultures revealed AM fungal species that were not detected in the control. The abundance of AM fungal spores showed a weak correlation with soil chemical properties. Additionally, the Maize variety proved to be a more effective host for propagating AM fungal spores compared to the other crops evaluated. These findings suggest that further research is needed to determine whether the co-culturing of multiple traditional host plants is as effective as the monoculture cultivation of a single host plant for AM fungi propagation.

## 1. Introduction

Traditional crops are cultivated worldwide [1]. In Africa, they form a vital part of the region’s rich biodiversity heritage and are essential components of local diets [2]. These crops contribute to agricultural diversification, support cultural traditions, and play a critical role in ensuring community food security [3]. Their agronomic potential enhances dietary diversity across both developing and developed countries [4,5].

Many traditional crops are resilient to harsh climatic conditions due to their strong adaptability to local environments [6]. This adaptability offers valuable opportunities to diversify and improve farming systems [7]. Small-scale farmers, particularly those in centers of crop diversity, play a crucial role in conserving those crops. By cultivating traditional varieties, they contribute to the ongoing process of crop evolution and maintain the genetic diversity essential to environmental changes [8].

Despite their importance, these traditional crops have often been marginalized in favor of commercial or non-traditional crops, primarily due to economic and market pressures [9]. The result has been the shrinking of dietary diversity, declining species diversity on farms, and the disappearance of indigenous ecological knowledge [10,11]. However, a growing interest in these crops is emerging, driven by social, environmental, and economic concerns [12]. This interest strongly supports the agroecological transition aimed at responding to food shocks and crises stemming from globalized food systems [13,14]. As a result, research has largely focused on their potential to enhance food security and preserve biodiversity for future generations. Yet there remains a significant gap in understanding the diversity and role of microbial communities in the rhizosphere of these crops.

Arbuscular mycorrhizal (AM) fungi form symbiotic associations with over 80% of essential plant species [15]. The emergence of AM fungi closely coincided with the evolution of vascular land plants around 450 million years ago [16], with the oldest fossils dating back to approximately 407 million years ago [17]. One key to the successful cultivation of traditional crops may lie in strengthening mutualistic partnerships with beneficial soil microbes such as AM fungi. Supporting this view, Cobb et al. [18] demonstrated that landraces respond more positively to mycorrhizal association than hybrids, particularly in low-fertility soil. Similarly, Karima et al. [19] reported that native AM fungi, being well adapted to local conditions, exhibit a higher colonization rate and facilitate more efficient nutrient uptake. In light of these findings, the co-adoption of traditional crop varieties and native AM fungi could be a promising strategy to enhance agricultural productivity. Although some studies indicate that functionally similar plant species tend to associate with the same or functionally similar AM fungi, other evidence suggests that plants with different functional traits harbor distinct AM fungal communities [20]. For this reason, AM fungi research and its practical use as a low-input technology on traditional crops is an interesting area to delve into.

Trap cultures are widely used and cost-effective traditional methods for the mass production of AM fungi in soil-based systems, employing a living host plant [21]. These cultures are also the preferred approach in taxonomic research, as they yield sufficient quantities of viable AM fungal spores [22,23], and mycorrhizal-root material for both scientific and practical applications [24]. However, the efficiency of trap cultures is influenced by a complex of interactions among the host plant genotype, environmental conditions, and the AM fungal species. Gaur and Adholey [25] demonstrated that the success of mycorrhizal propagation varies significantly with the type of host. Therefore, selecting an appropriate host plant is a critical factor in optimizing AM fungi sporulation, and a wide variety of host plant species have been employed in trap culture systems [26,27]. Given the context-specific nature of the AM fungi propagule, a deeper understanding of the host plant mechanisms that regulate AM fungal sporulation is essential for the development of effective AM fungal-based inoculum. In this study, we examine the influence of traditional host plants on AM fungal communities. Specifically, we hypothesized that both the host plant and soil origin shape the structure of AM fungal communities.

## 2. Materials and Methods

### 2.1. Study Area

The experiments were conducted at the Kenyatta University Teaching and Research Farm, located in the Agricultural Science and Technology Department, Kenyatta University (1.182210000° S, 36.920721667° E, 1720 m above sea level), in Kiambu County, Kenya. The area experiences a bimodal rainfall pattern, with short rainy season occurring from October to December, and the long rains falling between March and June. The annual average rainfall is approximately 850 mm. Kiambu has a mean monthly temperature ranging from 20 °C to 27 °C and relative humidity levels between 65 and 84%.

### 2.2. Soil Sampling, Treatment, and Analysis

**Figure 1:**
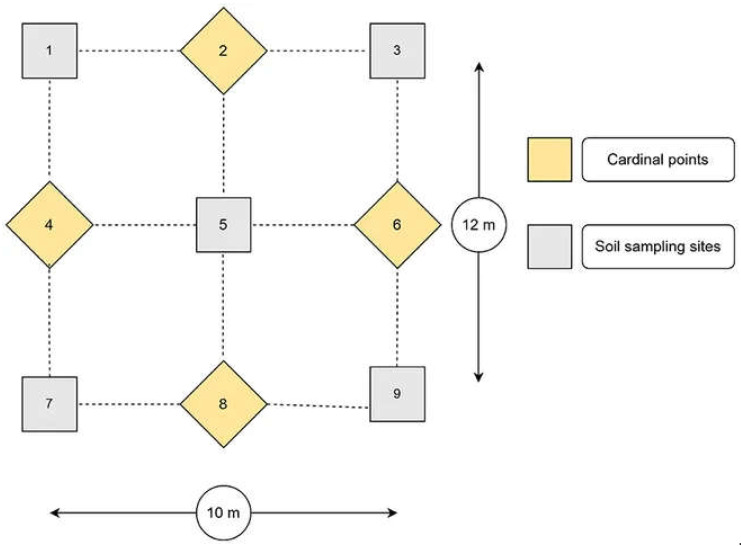
Overall soil sampling scheme per plot. The numbers 1, 3, 5, 7, and 9 represent the soil sampling points, while the numbers 2, 4, 6, and 8 represent the cardinal directions.

Soil samples were collected from 34 farmer-managed agricultural fields in Makueni County to establish trap cultures. Baseline soil samples were collected during the dry season of late September 2023, when root activities had declined and AM fungal spores were expected to be highly sporulating [28]. A soil sampling plot was demarcated, measuring 12 m by 10 m, in each of the 34 agricultural fields, as shown in (Figure 1). This was followed by collecting nine sub-samples (5 cm in diameter and 20 cm in depth) in each plot, following the procedure outlined in [29]. After removing any loose litter, the soil sampling proceeded by collecting the first sample from the central point (number 5). In addition, four sampling points were established, with 3 m measured in the cardinal directions 2 and 8 from the central point and 2.5 m measured in the cardinal directions 4 and 6 from the central point. A sample was collected at each of these points, resulting in a total of four samples. Furthermore, from the projected four corners (1, 3, 7, and 9), four soil samples were collected. The soil sub-samples from each plot were manually homogenized by mixing them in a clean bucket. A 1 kg sample was drawn from the composite sample, stored in a plastic bag, and labelled with the owner’s name, GPS coordinates, field history, and sampling date. Finally, 34 soil samples were collected for establishing trap cultures and AM fungi spore extraction as described under Section 2.4.

The area is characterized by smallholder mixed farming, where farmers engage in both crop and livestock production. Major crops cultivated include maize (*Zea mays*), pigeon peas (*Cajanus cajan*), sorghum (*Sorghum bicolor*), cowpeas (*Vigna unguiculata*), millet (*Eleusine coracana*), common beans (*Phaseolus vulgaris*), green grams (*Vigna radiata*), and mangoes (*Mangifera indica*) [30]. Soil fertility management practices among farmers in the area varied, using organic fertilizers, inorganic fertilizers, or a combination of both [30]. The soil samples were collected from the farmers’ fields who were selected during a co-design workshop implemented by a team of experts from the Agroecology Initiative of the Consultative Group on International Agricultural Research (CGIAR) [31].

Approximately, 200 g of the soil samples were then dried at room temperature and homogenized at the Kenyatta University Agricultural Laboratory. The samples were shipped and analyzed at the IITA International Institute for Tropical Agriculture (IITA) laboratory in Dar es Salaam. 10.0 g of dry soil was suspended in 25 mL of distilled water (soil:water ratio 1:2.5). The mixture was shaken by a mechanical shaker for 30 min to ensure thorough mixing, and then allowed to settle for 1 h. Electrical conductivity (EC), which reflects the concentration of soluble salts in the soil, was determined using an EC meter prob inserted in a supernatant (clear liquid above the settled particles) for EC readings. The EC values were measured with an electrical conductivity meter (Digital conductivity meter model 51-193 Spectronics India). Thereafter, the soil pH was measured using a pH meter (Hanna Instruments, Woonsocket, RI, USA), after calibration with buffer solutions of (4, 7, and 10) according to the method described by [32]. The soil samples were analyzed using the chromic acid titration method to estimate the total soil organic carbon (%) [33]. Total nitrogen (Ntot) was determined using the Kjeldahl method (KJELTEC 8200 Automatic Nitrogen Distiller unit_Foss, FOSS Analytical, Hillerød, Denmark) with an alkaline concentrated sulphuric acid digestion with catalyst of (Copper II sulphate, potassium sulphate, and selenium powder). For Available phosphorus (P) the M3 extraction, 3 g air-dried, 2 mm sieved soil was mixed with 30 mL of the Mehlich 3 extracting solution [34] and shaken for 5 min on an end-to-end orbital shaker. This was left to settle for 10 min and later centrifuged for 10 min at 3000 rpm. In the filtrate, Phosphorus readings were done using a UV-Vis spectrophotometer (Thermo Scientific-Genesys 10 uv_vis, Thermo Fisher Scientific, Waltham, MA, USA), while exchangeable bases (Calcium, Magnesium, and Potassium), micronutrients (Iron) were read using an atomic absorption spectrophotometer (Bulk Scientific Model 210 VGP, Buck Scientific Inc., East Norwalk, CT, USA).

### 2.3. Soil Chemical Properties

Soil chemical data are shown in Table 1. Statistical measures such as the mean, median, range, and standard deviation are provided in Table 2. The results revealed that the agricultural field soil was characterized by low to high inherent fertility.

#### 2.3.1. Soil pH

The soil pH values of the agricultural fields ranged from 6.3 to 7.2, with a mean of 6.7, showing that the soils were weakly acidic in the area.

#### 2.3.2. Electrical Conductivity

The electrical conductivity (EC) values observed in the agricultural fields were close to each other, with most of the fields recording EC values < 0.02, except field number 17, which recorded 0.03. The mean was 0.02, an indicator that the soils were non-saline, and had lower EC values than the recommended values of between 0.8 and 1.8 mhos/cm for plant growth.

#### 2.3.3. Total Organic Carbon

The total organic carbon (TOC) content in the soil across the agricultural fields revealed some variation, ranging from 0.76 to 1.94%, with a mean of 1.07. In general, the agricultural fields had lower values compared with the national threshold value of 1.74% SOC recommended as the critical concentration for sustaining crop production in low-input tropical soils [35].

#### 2.3.4. Total Nitrogen

The total nitrogen (TN) levels across the agricultural fields were generally very low since the TN ranged from 0.09 to 0.2% with a mean of 0.13. Most farms had low TN against the recommended values of 0.3 to 0.4%.

#### 2.3.5. Available Phosphorus

Available phosphorus (P) levels ranged from 2.8 mg/kg to 96.48 mg/kg, with an average of 21.67. This was lower than the average agronomic soil P threshold of 53.69 mg/kg Mehlich-3 P for all the crops as reported by [36]. This is a clear indicator that P application in most agricultural fields would result in a significant yield increase.

#### 2.3.6. Available Iron

The available iron (Fe) ranged between 35.83 mg/kg and 225 mg/kg with a mean of 102.39. The agricultural fields recorded much higher Fe limits than the reported optimum Fe level of 10 mg/kg for plant growth by [37].

#### 2.3.7. Exchangeable Ions

The exchangeable ions were as follows: Calcium (Ca) values ranged from 4.05 mg/kg to 25.71 mg/kg, with a mean of 10.54. Ca was extremely low as described by the grading system of [38]. The magnesium (Mg) values ranged from 0.62 Cmol+/kg to 3.42 Cmol+/kg, with a mean of 1.92. The Mg values ranged from low to high, as [39] rated exchangeable Mg as very low (<0.5), low (0.5–1.5), medium (1.5–3.3), high (3.3–8.3), and very high (>8.3 Cmol (+) kg^−1^) levels. The potassium (K) values ranged from 0.21 Cmol+/kg to 4.86 Cmol+/kg, with a mean of 0.85. The K was moderate to high based on the classification of [40]. Excess K should be avoided as it may lead to nutrient imbalances, especially affecting calcium and magnesium uptake.

### 2.4. Trap Culture Establishment

**Figure 2:**
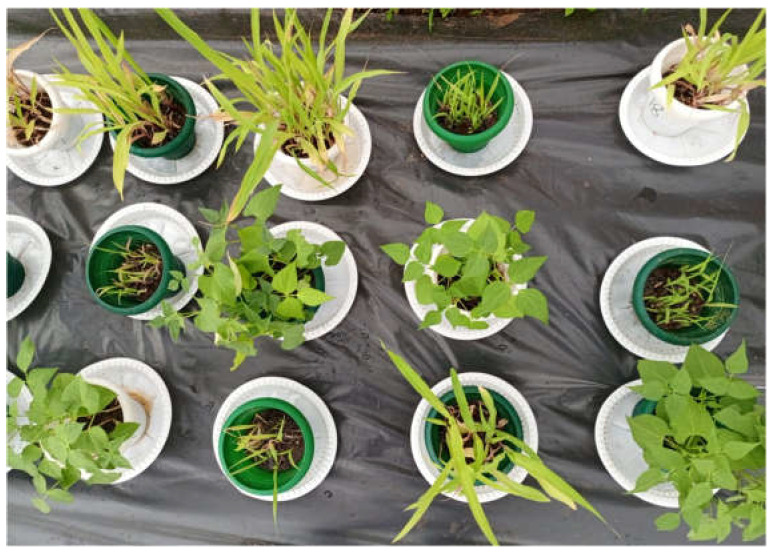
Two months of trap cultures established using maize, beans, and sorghum as host plants, and 34 agricultural field soils from the arid areas of eastern Kenya.

Trap cultures were established in September 2023 using fresh soil samples collected from agricultural fields. Soil from each of the 34 agricultural fields was mixed with autoclaved river sand (121 °C, 1 h) at a 1:1 (*v*:*v*) ratio and transferred into 1 L pots. Each pot was planted with one traditional host plant species: (1) Maize (*Z. mays* L.) cv, (2) Sorghum (*S. bicolor* (L) Moench), and (3) Common beans (*P. vulgaris*). Their local names within the area are: (1) Kinyaanya variety, (2) Vaasya variety, and (3) Katumbuka variety, respectively. These were some of the landraces grown in the soil sampling area. The seeds of each trap species were evenly sown per pot. The trap cultures were replicated three times and arranged in a completely randomized design inside the greenhouse (Figure 2). Pots were maintained under greenhouse conditions and watered as recommended. Two weeks prior to harvest, watering was stopped to allow the pots to dry in situ, thereby halting plant growth and promoting sporulation of AM fungal species.

### 2.5. Extraction and Quantification of AMF Spores

Spores of AM fungi were extracted from the trap culture after four months, while those from the agricultural field soil were extracted immediately by the wet-sieving and decanting method as described by [41], with modifications by [42]. Briefly, 50 g of the trap culture soil was suspended in 500 mL of tap water and stirred for one minute. The suspension was then sequentially sieved, running tap water through mesh sizes of 710, 400, 200, 100, and 45 μm to separate spores by size. The material retained on each sieve was collected into separate beakers. Spore suspensions were then transferred into 50 mL centrifugation tubes and centrifuged in a two-layer sucrose-water solution (20% and 60% *w*/*v*) for 5 min at 2700 rpm [43]. The supernatant was decanted into a 45-μm sieve, thoroughly rinsed with tap water, and transferred to Petri dishes for quantification under a stereomicroscope. First, different morphotypes were initially separated in water under a dissecting microscope based on spore color and size. Then, the representative of each morphotypes were mounted on microscope slides using polyvinyl–lactic acid–glycerol (PVLG) and a 1:1 (*v*/*v*) mixture of PVLG with Melzer’s reagent (Atom Scientific, Hyde, UK) [44] to observe wall AM fungi structures and other distinguishing features under a compound microscope at 40× magnification (Zeiss Primostar 3 microscope, Carl Zeiss, Jena, Germany).

The isolated morphotypes were classified into either known species or types that could not be placed in a current species, based on [45] description key, including spore colour, size, surface ornamentation, hyphal attachment, reaction to Melzer’s reagent, and wall structure. Identification was also carried out using online species descriptions from the International Culture Collection of Vesicular–Arbuscular Mycorrhizal Fungi (INVAM) (http://fungi.invam.wvu.edu/the-fungi/species-descriptions.html, accessed on 6 July 2025), descriptions in the primary literature, and experience of the authors.

### 2.6. The Occurrence, Relative Abundance, and Dominance of AM Fungal Morphospecies Structure

The isolation frequency (IF) of occurrence was calculated as the percentage of samples in which a genus or species occurred among all samples, and it reflects the distribution status.

Relative abundance (RA) = (the spore of AM fungi of a genus or species in the samples/the total AM fungi spore in the sample) × 100%.

Importance value (IV) = (IF + RA)/2. The IV > 50% was considered as a dominant, 30–50% as very common, 10–30% as common, and 10% as rare species [46].

### 2.7. Statistical Analysis

All statistical analyses were performed in R (version 4.5.0). Data were first tested for normality using the Shapiro–Wilk test, and homogeneity of variance was assessed with Levene’s test. When assumptions were not met logarithmic transformation was applied before analysis. A one-way analysis of variance (ANOVA) was used to evaluate significant differences in spore abundance among the host plants and the agricultural field soils, followed by Tukey’s post-hoc test for pairwise comparisons (*p* < 0.05). The structure of the AM fungal community was characterized using ecological diversity metrics, including isolation frequency, relative abundance, and importance value as described under Section 2.6. To examine the relationships between AM fungi spore abundance and soil chemical properties, principal component analysis (PCA) and Pearson correlation test were conducted.

## 3. Results

### 3.1. AM Fungal Genera Composition

**Figure 3:**
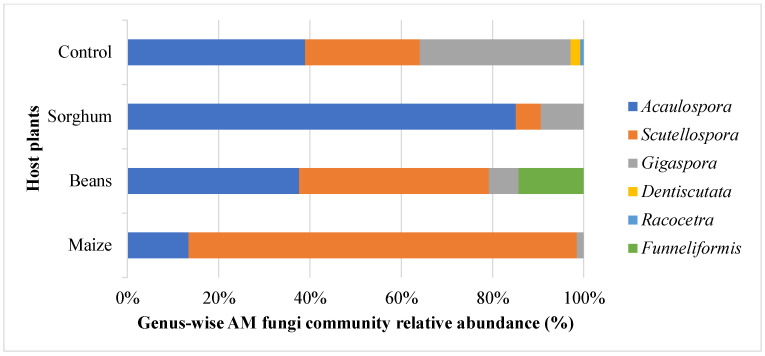
Genus-wise taxonomic profile composition of native arbuscular mycorrhiza fungal communities’ relative abundance in maize, beans, sorghum, and control (agricultural field soil).

Across all three host plants, six AM fungal genera were detected. *Scutellospora* and *Acaulospora* were the dominant genera, accounting for more than 80% of the genera in maize and sorghum trap cultures (Figure 3). Compared to the control (agricultural field soil), the relative abundance of these two genera was also higher. The bean variety induced four genera: *Acaulospora*, *Scutellospora*, *Gigaspora*, and a unique genus, *Funneliformis*, compared to maize and sorghum, which had slightly lower relative abundances. These genera belonged to six AM fungal families: Gigasporaceae (*Gigaspora*), Scutellosporaceae (*Scutellospora*), Dentiscutataceae (*Deniscutata*), Racocetraceae (*Racocetra*), Acaulosporaceae (*Acaulospora*), and Septoglomeraceae (*Funneliformis*).

### 3.2. Shared and Unique AM Fungal Genera

**Figure 4:**
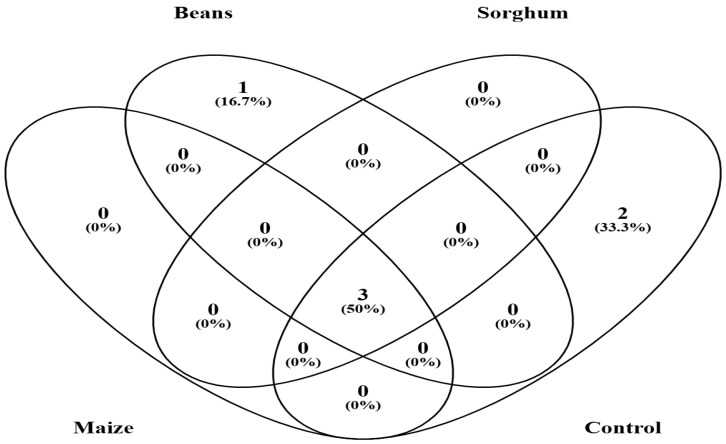
Venn diagram analyses of AM fungal genera in trap cultures and field soil samples. A Venn diagram demonstrated the numbers of shared and unique observed AM fungal genera at 100% similarity among the samples [47].

The Venn diagram analysis showed that the three hosts and the control (agricultural field soil) shared three genera at the genus level, accounting for 50% of the total genera observed (Figure 4). The shared genera primarily belonged to *Acaulospora*, *Scutellospora*, and *Gigaspora*. Interestingly, the number of unique genera was detected in the field soil at 33.3% and in beans at 16.7%. These unique genera were *Dentiscutata* and *Racocetra* in the control, and *Funneliformis* in the bean variety.

### 3.3. Arbuscular Mycorrhiza Fungal Species Diversity

Morphological identification of freshly formed AM fungal spores in trap cultures recovered 32 species, whereas the control had 26 species (Table 3). Maize host plant recorded 26, Sorghum 19, and beans 12 species, respectively. Overall, the AM fungal species with the highest proportion of 77% was *Scutellospora* sp 3 in the maize host plant. In sorghum, the highest proportion of 52% was in *Acaulospora delicata*, while in beans, the highest proportion of 31% was in *Scutellospora dispurpurascens*. In the control, the highest proportion of 25% was observed in *Acaulospora denticulata*. Strikingly, 12 species in the control were not propagated in trap cultures. Also, 19 species detected in trap cultures were not isolated in the control.

### 3.4. The Abundance and Distribution of Fungal Genera’s Spores per Host Plant

**Figure 5:**
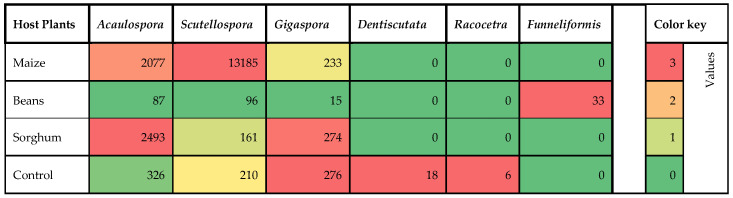
Heatmap showing the spore abundance of the six dominant AM fungal genera isolated from maize, beans, and sorghum trap cultures and control (agricultural field soil). The color of each heatmap rectangle indicates the spore abundance of each genus isolated across plant species and control, as shown in the legend.

We visualized the distributions of the genera in a heatmap covering all the studied host plants and the control (agricultural field soil) (Figure 5), indicating the genera and their cumulative number of spore abundance per host plant. Most (13,185) of these frequent fungal genera were *Scutellospora* in the maize variety, (2493) were *Acaulospora* in the sorghum variety, and (33) were *Funneliformis* in the bean variety.

### 3.5. Isolation Frequency and Importance Value

The isolation frequency (IF) of each species is depicted in Table 4. Genus *Acaulospora* was found to have the highest isolation frequency (62%) and (56%) in maize and sorghum host plants, respectively, while *Scutellospora* had the highest isolation frequency (21%) in bean host plants. Interestingly, the genus *Gigaspora* had the highest IF of (56%) in the control (agricultural field soil). The important value (IV) of the genera of AM fungi is also shown in Table 4. The most dominant genus in maize and bean host plants was *Scutellospora*, scoring (50%) and (31%), respectively. Conversely, the most predominant genus in sorghum was Acaulospora (71%), while in the control, the most predominant genus was Gigaspora (44%). However, not every dominant genus had both high IF and IV with the same host plant. *Acaulospora* had high IF in maize and sorghum, but was dominant in sorghum alone. Likewise, *Scutellospora* had high IF in beans but was dominant in both beans and maize.

### 3.6. Arbuscular Mycorrhiza Spore Abundance

#### 3.6.1. Occurrence of AM Fungal Spore Abundance (50 g Soil) as Influenced by the Agricultural Fields

The spore abundance of AM fungi obtained from trap cultures differed significantly based on the agricultural field and each host plant (Table 5). Among the leading spore abundance, the maize variety recorded a total of 4045 spores in field number six, the bean variety 20 spores in field number 33, while in sorghum variety recorded 367 spores in field number 20.

#### 3.6.2. Occurrence of AM Fungal Spore Abundance (50 g Soil) as Influenced by Host Plants

The variation in AM fungal spore abundance among the host plants was statistically significant (Table 6). The highest spore abundance was seen for maize across 15 agricultural fields, followed by sorghum variety across 9 agricultural fields, while bean variety lagged with none. The highest spore abundance of 4045 was recorded in agricultural field number six (F6).

#### 3.6.3. Cumulative AM Fungal Spore Abundance in Different Host Plants

The mean cumulative AM fungal spore abundance of the host plants ranged from 5152 to 77 (Table 7). The highest spore abundance was observed in the maize variety, followed by the sorghum variety. In contrast, the mean spore abundance for the bean variety as a host plant was significantly lower, recording the least number of spores compared to the control (agricultural fields).

### 3.7. Relationship Between AM Fungal Spore Abundance Composition, Soil Physicochemical Properties, and Agricultural Field Soils

**Figure 6:**
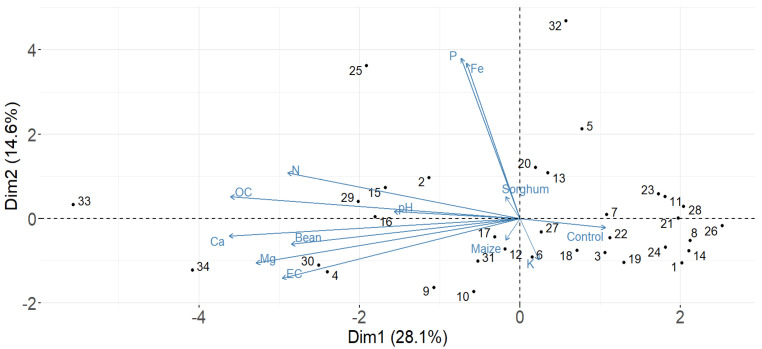
The principal component analysis (PCA) reveals the relationship between AM fungal spore abundance and soil chemical properties in relation to the agricultural field soils. The X and Y axes are the first and second principal components, respectively.

**Figure 7:**
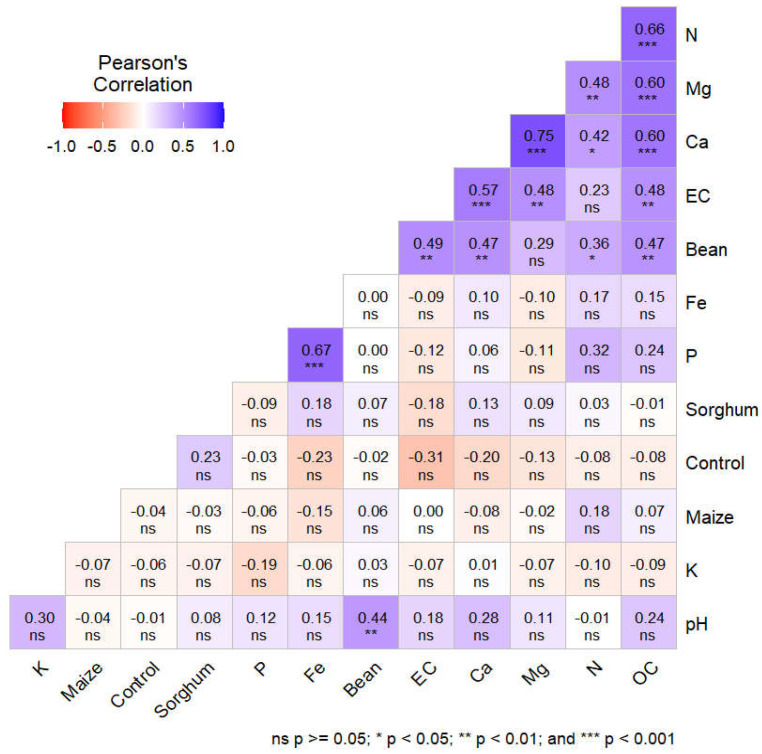
Pearson correlation coefficients between host plants, AM fungal spore abundance, and soil chemical properties.

To examine the AM fungal spore abundance in response to soil chemical properties among 34 agricultural field soils, a principal component analysis (PCA) was performed (Figure 6). In addition, the Pearson correlation test among all the measurements was calculated (Figure 7). The first two principal components (PCs) captured 43% of the trait variation (PC1: 28%; PC2: 15%). The AM fungal spore abundance of sorghum, maize, and the control (agricultural field) had a weak correlation with soil chemical properties; AM fungal spore abundance of beans had a moderate correlation with soil chemical properties, especially total N, Mg, and EC (Figure 6). On the other hand, the Pearson correlation coefficient between spore abundance and the soil chemical properties elicited a very weak correlation (Figure 7). Notably, the available P, K, and spore abundances from all the host plants and the control had a very weak correlation, which was not significant. However, most of the chemical parameters showed a slight, weak positive or negative correlation with spore abundance, although this varied based on the host plant. Additionally, spore abundance in the control was negatively correlated with all the parameters, which was not significant, while some of the parameters, such as total N, pH, Ca, EC, and total OC, had a slightly significant positive correlation with spore abundance from bean trap cultures.

## 4. Discussion

Previously, we reported on the diversity and abundance of AM fungal spores in semi-arid lands and their key drivers [29]. This study investigated the diversity and abundance of AM fungal spores from trap cultures established with precise soil samples from the same region, using three traditional plants as host plants. As expected, host plants differed in their capacity to propagate AM fungal genera and species (Figure 3 and Figure 4, and Table 3). Most importantly, the AM fungal species exhibited distinct patterns of host preference, such that an individual host plant emerged as a better host for specific AM fungal species. For instance, the genus *Scutellospora* was strongly linked to maize variety, whereas *Acaulospora* was associated with Sorghum variety (Figure 3 and Figure 4). Specifically, a higher number of *Scutellospora* sp 3 was detected in the maize host plant, *Acaulospora delicata* in sorghum, and *Scutellospora dispurpurascens* in beans. It is apparent that host plants actively influence the AM fungal composition and structure [67]. This is through differential reproduction (e.g., sporulation rates), growth, or survival of different fungal taxa [68,69]. One underlying mechanism might be that the host plants developed divergent root morphologies and allocated photosynthates preferentially to the AM fungi partners, just as reported previously by [70,71]. Concordantly, [72,73] revealed that while AM fungal species are generalists, there is a degree of preference in their association with host plants. The host plants can filter AM fungal communities directly [74]. Furthermore, multiple cases of evidence from Trejo-Aguilar et al. [75] and Yang et al. [76] indicate that certain host plants show preferences for certain AM fungi. Alternatively, plant hosts could impart changes in the soil environment (e.g., pH, carbon) that alter AM fungal communities indirectly [77].

There was a huge discrepancy between the AM fungal genera and species isolated from a direct analysis of initial agricultural field soil (control) and their corresponding trap cultures. On one hand, two AM fungal genera, *Dentiscutata* and *Racocetra*, and their corresponding species, as reported in the initial agricultural field soil samples (control), were absent in the trap cultures (Figure 3 and Table 3), respectively. On the other hand, the composition of the AM fungal species in trap cultures varied from that of the control (Table 3). Strikingly, 12 species in the control were not propagated in trap cultures. As well, 19 species detected in trap cultures had not been isolated from the control (Table 3). Interestingly, a unique genus, *Funneliformis*, was observed in the bean trap culture (Figure 3, Figure 4 and Figure 5). The findings were also supported by the dominance of some AM fungi genera in the trap cultures as influenced by different host plants (Table 4). One plausible explanation for these findings would be that the AM fungi species require specific plant hosts to sporulate. Such characteristics corroborate those reported by Stutz & Morton [78], who demonstrated that, compared to the first trap subculture, only 75% of the original species were found after three trap culture cycles. The findings also align with those of Oehl et al. [79], who noted that many of the AM fungi commonly found in extensive grasslands and maize fields were not recovered in the trap cultures. Furthermore, Tchabi et al. [80], reported a total of 60 AM fungal species, with only seven species sporulating in the trap cultures. This can be explained by other studies, which indicated that host plant species affect fungal development in trap cultures [81,82]. However, the absence of sporulation in the first trap culture cycle does not indicate the absence of the fungal organism. Probably, additional cycles of trap cultures are required for AM fungi to begin to sporulate sufficiently. Our study was not the first to point out this, since Tenzin [27] reported that the first trap culture failed to increase AM fungi spore density on maize and sorghum roots, although the host plants were colonized fully by other fungi and moderately by AM fungi.

Another intriguing pattern was in the AM fungi spore abundance variation based on the host plants. Notably, we obtained compelling insights into AM fungi sporulating abundantly in a sample that had registered zero spores from direct analysis of the initial agricultural field soil, and vice versa (Table 5 and Table 6). In this case, the host-plant dataset provided a snapshot of host dependence, with maize variety being a more efficient host plant, thus recording a cumulative high number of spores than the other hosts (Table 7). This is a clear indicator of host-dependent processes in trap culture systems. The results imply that in cases of high spore abundance, the host plant triggered the sporulation of more tolerant species to the new growth conditions, consistent with [44,83]. The findings of host dependence broadly match the result by Bever [84], and by other published studies [27,85,86], which observed significant variation in AM fungi sporulation in different plant hosts. The mechanism of this host-dependent sporulation was not investigated, but it could result directly from host-dependent growth of the fungi, host root growth characteristics, or indirectly through host-dependent alteration of the soil environment, and through host-mediated interactions of individual AM fungal species with each other or with other components of the soil community. Furthermore, the monocots performed better than the legumes. Indeed, another study by Al-Raddad [87] found the highest spore population with cereal plants (barley and maize), followed by chickpea and beans. The explanatory power of this is evidenced by [88], who stated that from living host plants, monocots and plants with extensive root systems are very good hosts for the propagation of AM fungi.

Lastly, this study examined the relationship between spore abundance and soil chemical properties, and it was clear that the soil characteristics also shaped the AM fungi sporulation in trap cultures. Our main findings indicated that the AM fungi spore abundance of sorghum, maize, and the agricultural field (control) had a weak correlation with soil chemical properties; while spore abundance of beans had a moderate correlation with soil chemical properties, especially total N, Mg, and EC (Figure 6). This highlights the main role played by host plants in altering the chemical properties of the trap culture media, which could be through root exudates. As well, soil properties have a considerable influence on AM fungi [89]. This reflects results from Koorem et al. [90] and Burke et al. [91] who reported that AM fungi spore abundance and community diversity were positively related to soil nitrogen. Pearson correlation further revealed that P and K were negatively correlated with spore abundance (Figure 7). According to García-González et al. [92], the physicochemical characteristics of soils can generate variations in the number of spores. Our findings agree to a large extent with those of [89,93] on soil P, which was reported to be negatively correlated with spore numbers. On the other hand, the K was moderate to high, and this could have led to it influencing the spore abundance negatively. It is worth noting that the spore abundance in the control was negatively correlated with all the soil chemical parameters, while some of the parameters, such as N, pH, Ca, Mg, EC, and OC, had a slight positive correlation with spore abundance in bean trap cultures, this shows that they were good predictors of changes in AM fungi spore abundance in trap cultures.

## 5. Conclusions

Our comparative approach revealed that traditional host plants have a substantial impact on the contemporary production of AM fungal spores. Each host plant and agricultural field soil varied in the AM fungal communities they support; therefore, these crops warrant priority for future studies as hosts for propagating AM fungal propagules. Furthermore, these hosts could be used strategically as ecosystem-specific host plants for preserving highly responsive and locally adapted AM fungal strains. To maintain biodiversity, we propose that further research needs to be done to determine whether co-culturing multiple traditional host plants and their respective trap cycles matches the effectiveness of a single-plant monoculture.

## Figures and Tables

**Table 1:** Chemical soil properties of agricultural fields.

Field No.	pH	EC (mhos/cm)	TOC (%)	TN (%)	P (mg/kg)	Fe (mg/kg)	Ca (mg/kg)	Mg (Cmol+/kg)	K (Cmol+/kg)
1	6.86	0.013	0.78	0.09	11.56	84.44	8.70	0.85	4.86
2	7.05	0.013	1.38	0.13	36.01	115.24	15.59	2.29	0.55
3	6.31	0.018	1.12	0.11	9.05	68.89	9.92	1.18	0.32
4	6.78	0.020	1.30	0.13	6.70	89.52	15.99	2.75	0.90
5	6.67	0.020	0.94	0.09	68.01	150.79	9.51	1.24	0.30
6	6.62	0.017	1.14	0.15	14.07	69.52	6.48	1.70	0.48
7	6.72	0.020	0.92	0.09	14.41	127.30	7.09	1.32	0.56
8	6.76	0.014	0.94	0.08	6.37	85.40	5.26	1.00	0.24
9	6.58	0.023	1.56	0.13	2.85	58.10	10.32	2.92	1.15
10	6.87	0.020	1.30	0.13	3.85	35.87	8.70	2.83	0.59
11	6.93	0.015	0.76	0.09	28.64	104.13	5.26	0.96	0.20
12	6.82	0.016	1.24	0.14	8.04	73.97	8.91	2.48	1.03
13	6.77	0.019	1.04	0.11	43.72	126.03	7.29	1.40	0.64
14	6.54	0.014	0.94	0.11	11.89	56.83	4.25	1.32	0.29
15	6.71	0.021	1.20	0.13	32.50	153.02	17.81	3.41	0.67
16	6.61	0.022	1.40	0.17	16.75	119.37	12.15	2.69	1.06
17	6.72	0.028	0.92	0.13	21.61	103.17	8.10	1.84	1.05
18	6.67	0.022	0.60	0.08	14.07	103.81	12.55	1.91	0.48
19	5.99	0.020	0.98	0.15	3.35	56.51	5.87	1.29	0.19
20	6.70	0.015	0.96	0.12	12.40	154.60	11.94	1.99	0.48
21	6.71	0.018	0.46	0.12	17.42	96.19	4.86	0.77	0.44
22	6.72	0.014	1.08	0.13	20.10	69.52	8.70	2.03	1.43
23	6.65	0.012	0.66	0.13	20.60	107.30	6.48	1.28	0.21
24	6.52	0.013	0.40	0.13	5.36	75.87	6.68	1.76	0.23
25	6.61	0.013	1.50	0.20	96.48	142.22	14.98	2.58	0.46
26	6.93	0.011	0.50	0.11	8.21	116.83	4.05	0.68	3.82
27	6.86	0.015	1.10	0.13	6.87	91.43	7.69	2.01	0.31
28	6.59	0.013	0.70	0.10	32.33	84.13	4.66	1.41	0.48
29	6.67	0.022	1.60	0.14	14.91	154.60	16.19	2.87	1.07
30	6.57	0.020	1.26	0.15	5.86	85.08	28.74	3.42	0.67
31	6.58	0.019	0.86	0.13	10.22	92.38	13.56	3.30	1.53
32	6.62	0.011	1.32	0.16	73.03	225.40	4.86	0.62	0.17
33	7.16	0.029	1.94	0.20	37.69	120.95	19.43	2.55	1.34
34	7.14	0.032	1.52	0.14	21.94	82.86	25.71	2.53	0.80

**Table 2:** The mean, median, range, and standard deviation of the soil chemical parameters.

Chemical Parameter	Mean	Median	Range	Std Dev
pH	6.7	6.71	1.17	0.22
EC	0.02	0.02	0.02	0.01
Total organic Carbon (%)	1.07	1.06	1.54	0.35
Total Nitrogen (%)	0.127	0.13	0.12	0.03
Phosphorus (mg/kg)	21.67	14.24	93.63	21.33
Iron (mg/kg)	102.39	94.29	189.53	37.62
Calcium (mg/kg)	10.54	8.70	24.69	5.97
Magnesium (Cmol+/kg)	1.92	1.88	280	0.82
Potassium (Cmol+/kg)	0.85	0.56	4.69	0.97

**Table 3:** Number of arbuscular mycorrhizal fungal species in trap cultures characterized under the host plants and control per 200 g of soil.

		Sorghum		Maize		Beans		Control		
No	Species	Spores Number	%	Spore Number	%	Spore Number	%	Spores Number	%	Reference
1	*Acaulospora delicata*	2353	51.79	1098	5.24	12	3.91	17	2.11	Walker and Sanders (1986) [48]
2	*Gigaspora gigantea*	510	11.23	164	0.78	12	3.91	0	0.00	Nicolson and Gerdemann (1968) [49]
3	*Gigaspora margarita*	438	9.64	80	0.38	0	0.00	156	19.35	Bentivenga and Morton (1995) [50]
4	*Acaulopora sp 4*	266	5.86	346	1.65	0	0.00	0	0.00	-
5	*Acaulopora sp 5*	240	5.28	50	0.24	0	0.00	0	0.00	-
6	*Acaulopora sp 2*	160	3.52	20	0.10	0	0.00	3	0.37	-
7	*Scutellospora calospora*	150	3.30	0	0.00	0	0.00	4	0.50	Walker and Sanders (1986) [51]
8	*Gigaspora albida*	112	2.47	12	0.06	0	0.00	42	5.21	Schenck and Smith (1982) [52]
9	*Gigaspora decipiens*	82	1.80	48	0.23	17	5.54	2	0.25	Hall and Abbott (1984) [53]
10	*Acaulospora rugosa*	48	1.06	20	0.10	0	0.00	0	0.00	Schenck et al. (1984) [54], Morton (1986) [55]
11	*Acaulospora sp 1*	40	0.88	164	0.78	8	2.61	0	0.00	-
12	*Acaulopora sp 3*	34	0.75	16	0.08	0	0.00	0	0.00	-
13	*Scutellospora dispurpurascens*	32	0.70	1290	6.16	96	31.27	0	0.00	Morton and Koske (1988) [56]
14	*Gigaspora sp 2*	28	0.62	0	0.00	0	0.00	15	1.86	-
15	*Gigaspora sp 1*	20	0.44	6	0.03	9	2.93	39	4.84	-
16	*Acaulopora sp 3*	12	0.26	16	0.08	0	0.00	0	0.00	-
17	*Scutellospora sp 1*	10	0.22	8	0.04	2	0.65	50	6.20	-
18	*Gigaspora sp 3*	4	0.09	0	0.00	0	0.00	0	0.00	-
19	*Gigaspora sp 4*	4	0.09	0	0.00	0	0.00	0	0.00	-
20	*Scutellospora sp 3*	0	0.00	16,180	77.25	6	1.95	0	1.49	-
21	*Acaulospora sp 7*	0	0.00	420	2.01	0	0.00	0	0.00	-
22	*Acaulospora sp 8*	0	0.00	372	1.78	0	0.00	0	0.00	-
23	*Acaulospora sp10*	0	0.00	370	1.77	0	0.00	0	0.00	-
24	*Scutellospora sp 6*	0	0.00	200	0.95	0	0.00	0	0.00	-
25	*Acaulospora sp 1*	0	0.00	164	0.78	0	0.00	25	3.10	-
26	*Acaulospora sp 9*	0	0.00	30	0.14	0	0.00	0	0.00	-
27	*Scutellospora sp 5*	0	0.00	22	0.11	0	0.00	82	10.17	-
28	*Acaulospora sp 6*	0	0.00	16	0.08	0	0.00	0	0.00	-
29	*Scutellospora sp 2*	0	0.00	9	0.04	15	4.89	31	3.85	-
30	*Scutellospora sp 4*	0	0.00	4	0.02	8	2.61	22	2.73	-
31	*Acaulospora scrobiculata*	0	0.00	0	0.00	78	25.41	0	0.00	Spain (1992) [57]
32	*Funneliformis sp*	0	0.00	0	0.00	44	14.33	0	0.00	-
33	*Acaulospora denticulata*	0	0	0	0	0	0	198	24.50	Sieverding and Toro (1987) [58]
34	*Acaulospora koskei*	0	0	0	0	0	0	32	3.97	Blaszkowski J. (1990) [59], (1995) [60]
35	*Acaulospora laevis*	0	0	0	0	0	0	19	2.36	Beilby (1980) [61]
36	*Acaulospora colombiana*	0	0	0	0	0	0	15	1.86	Spain (1992) [57]
37	*Dentiscutata scutata*	0	0	0	0	0	0	8	0.99	Walker and Diederichs (1989) [62]
38	*Dentiscutata heterogama*	0	0	0	0	0	0	8	0.99	Oehl et al. (2008) [63]
39	*Gigaspora rosea*	0	0	0	0	0	0	7	0.87	Bentivenga and Morton (1995) [50]
40	*Racocetra castanea*	0	0	0	0	0	0	6	0.74	Oehl et al. (2008) [63]
41	*Acaulospora rehmii*	0	0	0	0	0	0	5	0.62	Spain (1992) [57], Sieverding and Toro (1987) [58]
42	*Acaulospora tuberculata*	0	0	0	0	0	0	4	0.50	Janos and Trappe (1982) [64]
43	*Dentiscutata nigra*	0	0	0	0	0	0	3	0.37	Oehl et al. (2008) [63], Redecker (2013) [65]
44	*Dentiscutata biornata*	0	0	0	0	0	0	1	0.12	Goto et al. (2012) [66]

**Table 4:** Isolation frequency (IF) and importance value (IV) of AM fungal genera for the host plants.

	Maize		Beans		Sorghum		Control	
Genera	IF (%)	IV (%)	IF (%)	IV (%)	IF (%)	IV (%)	IF (%)	IV (%)
*Acaulospora*	61.76	37.58	14.71	26.18	55.88	70.51	41.18	40.09
*Scutellospora*	14.71	49.90	20.59	31.07	14.71	10.10	38.24	31.68
*Gigaspora*	38.24	19.87	14.71	10.60	44.12	26.74	55.88	44.45
*Dentiscutata*	0.00	0.00	0.00	0.00	0.00	0.00	8.82	5.49
*Rocacetra*	0.00	0.00	0.00	0.00	0.00	0.00	2.94	1.83
*Funneliformis*	0.00	0.00	2.94	8.61	0.00	0.00	0.00	0.00

**Table 5:** Mean spore abundance (mean ± SE) of arbuscular mycorrhizal fungi 50 g^−1^ dry soil in native dryland soils and host plants.

Agricultural Fields	Control	Maize	Beans	Sorghum
18	0.00 ± 0.0 ^p^	14.0 ± 4.0 ^hijk^	2.0 ± 0.0 ^def^	4.0 ± 2.0 ^ijk^
30	1.00 ± 0.0 ^op^	112.0 ± 4.5 ^d^	0.6 ± 0.5 ^fg^	47.0 ± 3.0 ^f^
29	1.00 ± 0.0 ^op^	4.0 ± 2.0 ^k^	1.0 ± 0.0 ^fg^	0.0 ± 0.0 ^k^
33	1.67 ± 0.5 ^nop^	373.0 ± 9.5 ^b^	20.3 ± 0.5 ^a^	131.0 ± 2.0 ^b^
16	1.67 ± 0.5 ^nop^	6.6 ± 3.5 ^jk^	3.6 ± 0.5 ^cd^	5.0 ± 1.0 ^ijk^
9	2.67 ± 0.5 ^mno^	143.0 ± 2.5 ^c^	0.0 ± 0.0 ^g^	59.7 ± 5.03 ^e^
3	2.67 ± 0.5 ^mno^	8.67 ± 2.5 ^ijk^	1.0 ± 1.0 ^fg^	1.0 ± 0.0 ^k^
20	3.33 ± 0.5 ^lmn^	62.0 ± 3.6 ^e^	0.0 ± 0.0 ^g^	367.0 ± 4.5 ^a^
17	3.67 ± 0.5 ^klmn^	0.6 ± 0.5 ^k^	1.0 ± 1.0 ^fg^	2.0 ± 0.^0 jk^
13	3.67 ± 0.5 ^klmn^	0.0 ± 0.0 ^k^	3.0 ± 0.0 ^cde^	2.6 ± 0.5 ^ijk^
1	4.00 ± 1.0 ^klm^	5.0 ± 1.0 ^k^	0.0 ± 0.0 ^g^	1.6 ± 0.5 ^k^
26	4.33 ± 0.5 ^klm^	4.0 ± 1.0 ^k^	0.0 ± 0.0 ^g^	5.6 ± 0.5 ^hijk^
10	4.33 ± 0.5 ^klm^	1.0 ± 1.0 ^k^	0.0 ± 0.0 ^g^	0.6 ± 0.5 ^k^
28	4.67 ± 0.5 ^jklm^	1.6 ± 0.5 ^k^	0.0 ± 0.0 ^g^	16.0 ± 9.0 ^gh^
21	4.67 ± 0.5 ^jklm^	36.7 ± 7.5 ^fg^	0.0 ± 0.0 ^g^	10.0 ± 3.0 ^hijk^
7	5.00 ± 1.0 ^ijkl^	3.0 ± 1.0 ^k^	0.6 ± 0.5 ^fg^	12.7 ± 0.5 ^hij^
2	5.00 ± 1.0 ^ijkl^	7.0 ± 1.0 ^jk^	4.0 ± 0.0 ^c^	68.7 ± 3.5 ^e^
24	5.33 ± 0.5 ^ijkl^	22.3 ± 3.2 ^ghi^	1.6 ± 0.5 ^efg^	8.6 ± 1.5 ^hijk^
6	5.67 ± 0.5 ^hijk^	4045.0 ± 5.0 ^a^	2.0 ± 1.0 ^def^	10.0 ± 3.0 ^hijk^
31	6.67 ± 0.5 ^hij^	10.7 ± 5.5 ^hijk^	1.33 ± 0.5 ^efg^	24.0 ± 4.0 ^g^
27	6.67 ± 0.5 ^hij^	25.0 ± 15.0 ^fgh^	1.6 ± 0.5 ^efg^	13.0 ± 7.0 ^hi^
25	7.00 ± 1.0 ^hi^	2.6 ± 0.5 ^k^	0.0 ± 0.0 ^g^	4.0 ± 1.0 ^ijk^
5	7.67 ± 0.5 ^gh^	3.6 ± 0.5 ^k^	0.0 ± 0.0 ^g^	4.6 ± 0.5 ^ijk^
34	9.67 ± 0.5 ^fg^	40.0 ± 7.0 ^f^	11 ± 0.0 ^b^	10.0 ± 2.0 ^hijk^
32	9.67 ± 0.5 ^fg^	21.0 ± 5.0 ^hij^	0.0 ± 0.0 ^g^	9.0 ± 0.0 ^hijk^
19	9.67 ± 0.5 ^fg^	5.6 ± 0.5 ^k^	0.0 ± 0.0 ^g^	1.6 ± 1.5 ^k^
12	9.67 ± 0.5 ^fg^	1.3 ± 1.1 ^k^	0.0 ± 0.0 ^g^	25.0 ± 9.0 ^g^
15	11.00 ± 0.5 ^f^	8.0 ± 2.0 ^ijk^	0.0 ± 0.0 ^g^	3.0 ± 3.0 ^ijk^
11	13.70 ± 0.5 ^e^	0.0 ± 0.0 ^k^	0.0 ± 0.0 ^g^	7.0 ± 2.0 ^hijk^
4	14.70 ± 0.5 ^e^	105.0 ± 5.0 ^d^	19.7 ± 1.53 ^a^	66.7 ± 0.5 ^e^
8	17.00 ± 1.0 ^d^	0.0 ± 0.0 ^k^	0.6 ± 0.5 ^fg^	5.0 ± 2.0 ^ijk^
23	21.50 ± 1.0 ^c^	76.0 ± 11.0 ^e^	2.0 ± 2.0 ^def^	89.0 ± 2.0 ^d^
14	34.50 ± 1.0 ^b^	2.0 ± 2.0 ^k^	0.0 ± 0.0 ^g^	5.6 ± 2.52 ^hijk^
22	61.30 ± 1.5 ^a^	2.0 ± 2.0 ^k^	0.0 ± 0.0 ^g^	118.0 ± 2.5 ^c^
*p* value	<0.05	<0.05	<0.05	<0.05

Values in each column with different letters are significantly different according to Tukey’s test at *p* < 0.05.

**Table 6:** Mean spore abundance of arbuscular mycorrhiza fungi as influenced by host plants and agricultural fields (50 g^−1^).

**Host Plants**	**F1**	**F2**	**F3**	**F4**	**F5**	**F6**	**F7**	**F8**	**F9**	**F10**	**F11**	**F12**	**F13**	**F14**	**F15**	**F16**	**F17**
Bean	0.0 ^a^	4.0 ^b^	1.0 ^b^	19.7 ^c^	0.0 ^c^	2.0 ^c^	0.6 ^c^	0.6 ^c^	0.0 ^c^	0.0 ^b^	0.0 ^c^	0.0 ^b^	3.0 ^a^	0.0 ^c^	0.0 ^b^	3.6 ^ab^	1.0 ^b^
Sorghum	1.6 ^b^	68.7 ^a^	1.0 ^b^	66.7 ^b^	4.6 ^b^	10.0 ^b^	12.7 ^a^	5.0 ^b^	59.7 ^b^	0.6 ^b^	7.0 ^b^	25.0 ^a^	2.6 ^a^	5.6 ^b^	3.0 ^b^	5.0 ^ab^	2.0 ^ab^
Control	4.0 ^a^	5.0 ^b^	2.6 ^b^	14.7 ^c^	7.6 ^a^	5.6 ^bc^	5.0 ^b^	17.0 ^a^	2.6 ^c^	4.3 ^a^	13.7 ^a^	9.6 ^b^	3.6 ^a^	34.0 ^a^	10.7 ^a^	1.6 ^b^	3.6 ^a^
Maize	5.0 ^a^	7.0 ^b^	8.6 ^a^	105.0 ^a^	3.6 ^b^	4045.0 ^a^	3.0 ^b^	0.0 ^c^	143.0 ^a^	1.0 ^b^	0.0 ^c^	1.3 ^b^	0.0 ^b^	2.0 ^bc^	8.0 ^a^	6.6 ^a^	0.6 ^b^
**Host Plants**	**F18**	**F19**	**F20**	**F21**	**F22**	**F23**	**F24**	**F25**	**F26**	**F27**	**F28**	**F29**	**F30**	**F31**	**F32**	**F33**	**F34**
Bean	2.0 ^b^	0.0 ^c^	0.0 ^c^	0.0 ^b^	0.0 ^c^	2.0 ^c^	1.6 ^c^	0.0 ^c^	0.0 ^b^	1.6 ^b^	0.0 ^b^	1.0 ^b^	0.6 ^c^	1.3 ^c^	0.0 ^c^	20.3 ^c^	11.0 ^b^
Sorghum	4.0 ^b^	1.6 ^c^	367.0 ^a^	10.0 ^b^	118.0 ^a^	89.0 ^a^	8.6 ^b^	4.0 ^b^	5.6 ^a^	13.0 ^ab^	16.0 ^a^	0.0 ^b^	47.0 ^b^	24.0 ^a^	9.0 ^b^	131.0 ^b^	10.0 ^b^
Control	0.0 ^b^	9.6 ^a^	3.3 ^c^	4.6 ^b^	61.0 ^b^	21.0 ^b^	5.3 ^bc^	7.0 ^a^	4.3 ^a^	6.6 ^ab^	4.6 ^ab^	1.0 ^b^	1.0 ^c^	6.6 ^bc^	9.6 ^b^	1.6 ^d^	9.6 ^b^
Maize	14.0 ^a^	5.6 ^b^	62.0 ^b^	36.7 ^a^	2.0 ^c^	76.0 ^a^	22.3 ^a^	2.6 ^bc^	4.0 ^a^	25.0 ^a^	1.6 ^b^	4.0 ^a^	112.0 ^a^	10.7 ^b^	21.0 ^a^	373.0 ^a^	40.0 ^a^

Values in each column with different letters are significantly different according to Tukey’s test at *p* < 0.05. F = agricultural fields.

**Table 7:** Mean cumulative AM fungal spore abundance.

Host Plants	Cumulative Spore Abundance
Beans	77.3 ± 2.8 ^d^
Control	303.0 ± 9.2 ^c^
Sorghum	1139.9 ± 49.5 ^b^
Maize	5152.0 ± 8.3 ^a^
*p* value	<0.05

Values in each column with different letters are significantly different according to Tukey’s test at *p* < 0.05.

## Data Availability

The raw data supporting the conclusions in this manuscript will be provided on request by the authors. The data is not publicly available due to the status of collaborative conformity.
